# Exercise improves gut microbial metabolites in an intensity-dependent manner: a pooled analysis of randomized controlled trials

**DOI:** 10.1080/19490976.2025.2579354

**Published:** 2025-11-30

**Authors:** Dejan Reljic, Hans Joachim Hermann, Walburga Dieterich, Markus Friedrich Neurath, Yurdagül Zopf

**Affiliations:** a Department of Medicine 1 – Gastroenterology, Pneumology and Endocrinology, University Hospital Erlangen, Friedrich-Alexander University Erlangen-Nürnberg, Erlangen, Germany; b Hector-Center for Nutrition, Exercise and Sports, Friedrich-Alexander University Erlangen-Nürnberg, Erlangen, Germany; c Deutsches Zentrum Immuntherapie, University Hospital Erlangen, Friedrich-Alexander University Erlangen-Nürnberg, Erlangen, Germany

**Keywords:** Exercise intensity, gut microbiota, interval training, metabolic diseases, metabolic syndrome, short-chain fatty acids

## Abstract

**Background:**

Obesity and the metabolic syndrome (MetS) are global health challenges. The gut microbiome, particularly its fermentation products, short-chain fatty acids (SCFAs), is increasingly recognized as a key modulator of cardiometabolic health. Growing evidence suggests that exercise may play a critical role in SCFA production. This study presents a pooled analysis of data from three randomized controlled trials to examine the effects of low-volume high-intensity (HIGH-EX) versus moderate-intensity (MOD-EX) interval training, each combined with single-set resistance training, on SCFAs and cardiometabolic health in obese MetS patients.

**Methods:**

Data from 113 patients allocated to 12 weeks of HIGH-EX, MOD-EX, or a control group (CON) were analyzed. Fecal SCFA concentrations, cardiorespiratory fitness (VO_2max_), muscle strength, inflammation (hsCRP), and MetS severity (MetS z-score) were assessed pre- and post-intervention. Exercise intensity was monitored via regular blood lactate measurements during interval training.

**Results:**

HIGH-EX and MOD-EX improved VO_2max_ (+4.2 and +2.7 mL/kg/min), strength across major muscle groups (+19 to 29%), hsCRP (–2.2 and –1.3 mg/L), and MetS z-score (–1.1 and –0.5 units). Only HIGH-EX significantly increased total fecal SCFAs (+30%), including acetate (+27%), propionate (+28%), and butyrate (+43%). Mean blood lactate concentrations during training were strongly correlated with SCFA increases (r = 0.68, *p* < 0.001). No changes were observed in CON.

**Conclusions:**

Low-volume combined interval and resistance training improved fitness and metabolic health at both moderate and higher intensities. However, only HIGH-EX enhanced SCFA production, potentially associated with lactate-mediated microbial adaptations. Exercise intensity may thus be a key determinant of gut-metabolic benefits.

## 1 Introduction

Obesity and the metabolic syndrome (MetS) have emerged as major global health challenges, contributing to increased morbidity and mortality rates worldwide.^1^^,^^2^ These conditions are closely linked to a spectrum of sequelae, including cardiovascular disease, type 2 diabetes, and several types of cancer.^3^^,^^4^

It is well established that the gut microbiota, comprising trillions of microorganisms residing in the gastrointestinal tract, plays an essential role in human metabolic health. Disruptions in the composition and function of the gut microbiota—commonly referred to as dysbiosis—have been increasingly associated with the development of obesity and MetS.^5^^,^^6^ While earlier studies primarily focused on microbial diversity and taxonomic shifts in obesity and related disorders, more recent research highlights the pivotal impact of microbial metabolites, particularly short-chain fatty acids (SCFAs), as critical mediators linking the gut microbiome to host metabolic health.^7^^,^^8^ SCFAs, primarily acetate, propionate, and butyrate, are produced through microbial fermentation of dietary fibers in the colon. These metabolites have been shown to exert various beneficial effects on host metabolism, including the modulation of lipid metabolism, improvement of insulin sensitivity, and maintenance of intestinal barrier integrity.^7^^,^^8^ Moreover, SCFAs have been reported to possess anti-inflammatory properties, which are crucial in mitigating chronic low-grade inflammation.^9^^,^^10^ Intriguingly, emerging evidence suggests that the profile and abundance of SCFAs may be even more critical for cardiometabolic health than the overall composition of the microbiota itself. For instance, alterations in SCFA levels, particularly a reduction in butyrate concentration, are frequently observed in individuals with obesity and MetS.^11^

Physical exercise is a cornerstone in the prevention and management of obesity and MetS.^12^^,^^13^ Beyond its well-established benefits on physical fitness, cardiometabolic status and body composition, it has been reported that exercise can enhance microbial diversity^14^ and promote the proliferation of SCFA-producing bacteria.^15^^,^^16^ However, human studies examining the effects of exercise on microbial metabolites in individuals with obesity or cardiometabolic disorders are still limited and yielded inconsistent findings.^17^ Liu et al.,^18^ for example, have reported increases in fecal propionate concentrations in overweight and obese males following 12 weeks of a combined high-intensity aerobic and resistance training. In contrast, two other trials did not find any effect of exercise on fecal SCFA concentrations in obese^19^ or type 2 diabetes patients.^20^ These discrepancies may stem from variations in study designs, including differences in training type, duration, and intensity. Notably, recent systematic reviews have suggested that particularly exercise intensity may play a crucial role in shaping microbiota-related outcomes, and it has been pointed out that more research is needed to explore these relationships.^21^^,^^22^ While previous research has provided evidence indicating superiority of higher- over moderate-intensity exercise for improving cardiorespiratory fitness (CRF),^23^ body fat content,^23^ and endothelial function,^24^ the effect of exercise intensity on microbial metabolites remains to be elucidated.

Apart from exercise intensity, the impact of so-called “low-volume” training modalities on SCFA profiles is still unexplored. These time-efficient exercise approaches, such as low-volume high-intensity interval training (LOW-HIIT)^25^ or single-set resistance training (1-RT),^26^ have gained increasing attention in both research and clinical contexts. Given that lack of time is a frequently reported barrier to regular physical activity among obese individuals,^27^ low-volume protocols are considered a viable alternative to traditional higher-volume exercise regimens. Importantly, growing evidence suggests that such protocols can induce significant improvements in CRF,^28-31^ body composition,^28-34^ inflammation,^28^ and cardiometabolic risk indices.^28-34^ However, there is still a lack of studies investigating whether low-volume exercise can modulate gut microbiota-derived metabolites.

To address these research gaps, the present study compared the effects of LOW-HIIT combined with progressive 1-RT exercise (HIGH-EX) versus low-volume moderate-intensity interval training (LOW-MIIT) combined with moderate-progressive 1-RT (MOD-EX) on fecal SCFA concentrations, cardiometabolic risk and physical fitness outcomes in a cohort of obese MetS patients. Based on previous research indicating the superiority of higher-intensity exercise in eliciting more favorable metabolic and anti-inflammatory adaptations,^23^^,^^24^^,^^33^ we hypothesized that HIGH-EX would produce greater improvements in fecal SCFAs, cardiometabolic health and physical fitness compared to MOD-EX.

## 2 Methods

### 2.1 Study design and patients

This manuscript represents a secondary analysis of data from three previous randomized controlled trials (RCTs).^32-34^ Patients in these trials were assigned either to HIGH-EX or MOD-EX performed over 12 weeks (trial 1, ClinicalTrials.gov ID: NCT03710447)^34^ or to a non-exercising control group (CON; trial 2, ClinicalTrials.gov ID: NCT03306069; and trial 3, ClinicalTrials.gov ID: NCT03306056).^32^^,^^33^ All patients received standard care nutritional counseling aimed at supporting dietary-induced weight loss through caloric restriction. Comprehensive descriptions of each trial’s methodology, including sample size determination, and randomization procedures, have been published previously.^32-34^ The data and results presented in this manuscript are original and have not yet been reported. The main outcomes for this study were SCFA concentrations in fecal samples. Secondary endpoints included cardiometabolic risk markers, anthropometric measurements, and indicators of physical fitness. In all trials, patients received comprehensive information regarding the study's objectives and procedures prior to enrollment and provided written informed consent in accordance with the ethical standards outlined in the Declaration of Helsinki. Ethical approval for the original study protocols was granted by the Medical Ethics Committee of Friedrich-Alexander University Erlangen-Nürnberg (approval numbers: trial 1: 132_18B, trial 2: 210_17B, and trial 3: 203_17B, respectively). Eligibility criteria have been described in detail elsewhere.^32-34^ In brief, eligible patients were required to be ≥18 years old, obese (BMI ≥ 30 kg/m²), diagnosed with MetS,^35^ and report a sedentary lifestyle. Additionally, inclusion in the current analysis required consent to provide an additional stool sample for SCFA analysis. Individuals were excluded if they were pregnant or had a clinical diagnosis of cardiovascular disease, cancer, or another major condition contraindicating safe participation in physical training. Only participants who completed a minimum of 80% of the prescribed exercise sessions were included in the final dataset.

### 2.2 Pre- and post-intervention assessments

Baseline assessments (T−1) were conducted ~1 week prior to the start of the intervention, including all study outcomes and medical clearance (ECG, blood, and urine tests) to ensure safe participation in the exercise programs. Patients arrived fasted, having avoided alcohol and strenuous activity for at least 24 hours beforehand. Post-intervention assessments (T−2) took place within one week following program completion, scheduled at similar times to control for circadian effects. Female participants were tested during the same menstrual phase as far as possible. Outcome assessors were blinded to group allocation.

#### 2.2.1 Blood pressure assessment

Upon arrival, patients were asked to empty their bladder and to rest seated for 5 minutes. Resting blood pressure (BP) was then measured twice on both arms (60-second interval) using an automated device (Omron M5 Professional, Mannheim, Germany). The arm with the higher average BP was used for analysis, and mean arterial blood pressure (MAB) was calculated as reported elsewhere.^36^

#### 2.2.2 Blood collection and biochemical analysis

Venous blood samples were obtained from an antecubital vein in a seated position. The samples were promptly transported to the Central Laboratory of the University Hospital Erlangen for biochemical analysis. Serum concentrations of glucose, triglycerides, low-density lipoprotein (LDL), and high-density lipoprotein (HDL) cholesterol were measured using automated photometric assays (AU700/AU5800 Clinical Chemistry Analyzer, Beckman Coulter, Brea, CA, USA), with intra-assay coefficients of variation (CV) ranging from 1.1–1.4%. A nephelometric assay (ATELLICA NEPH 630, Siemens, Erlangen, Germany; CV: 3.0%) was used to determine high-sensitivity C-reactive protein (hsCRP).

#### 2.2.3 Stool sample collection and analysis of short-chain fatty acids

Before and after the intervention, patients received standardized stool collection kits with written instructions. Additionally, verbal guidance was provided by trained study personnel to ensure compliance with the protocol. Samples were collected at home, stored cool (not frozen), and shipped promptly in insulated containers enclosed in a temperature-resistant, pre-labeled UN3373-compliant transport bag to the designated laboratory. Patients were specifically instructed to ship samples immediately after collection via courier delivery. Timely shipment was verified, as all samples were received by the laboratory within 24–48 hours of dispatch. Analysis was performed by GANZIMMUN Diagnostics GmbH (Mainz, Germany), an ISO/IEC 17025-accredited commercial laboratory. Upon arrival, samples were stabilized in 0.7 M HCl with 25% (w/w) NaCl, which halts microbial activity. According to the laboratory’s declaration, unprocessed stool samples are stable for ≥5 days in the sample tubes, and preserved aliquots are stable for ≥40 days when stored refrigerated or frozen. SCFA concentrations of acetate, propionate and butyrate were quantified by headspace gas chromatography-mass spectrometry (HS-GC-MS; Perkin Elmer Clarus 680, Clarus SQ 8S & Turbomatrix, Shelton, CT, USA), using 2-ethylbutyric acid as internal standard. Reported intra-assay coefficients of variation are 6.3% for acetate, 5.1% for propionate, and 4.0% for butyrate, indicating good analytical reproducibility.

#### 2.2.4 Assessment of anthropometric parameters and body composition

Waist circumference (WC) was assessed in standing position using a flexible tape (Seca, Hamburg, Germany). According to established guidelines,^37^ measurements were taken midway between the lower margin of the last palpable rib and the top of the iliac crest, and recorded to the nearest centimeter. Height was recorded to the nearest 0.1 cm with a calibrated stadiometer, and body weight was assessed using a seca mBCA 515 analyzer (Seca, Hamburg, Germany). This device was also used to assess body composition via segmental multi-frequency bioelectrical impedance, with proven validity and reliability in previous studies.^38^

#### 2.2.5 Calculation of metabolic syndrome severity score (MetS z-score)

The MetS z-score was calculated using sex-specific equations as previously reported by Johnson et al.,^39^ incorporating HDL, triglycerides, fasting glucose, WC, and MAB. By integrating these components into a single continuous metric, this composite score offers a more sensitive assessment of overall cardiometabolic risk than individual parameters alone.^40^

#### 2.2.6 Cardiopulmonary exercise testing (CPET)

Cardiorespiratory fitness was assessed via a symptom-limited ramp CPET protocol on a calibrated cycle ergometer (Corival cpet, Lode, Groningen, Netherlands). Initial workloads were set at 50 W, with 12.5 W/min (females) or 15 W/min (males) increases until volitional exhaustion. Heart rate was continuously monitored using a 12-lead electrocardiogram system (custo cardio 110, custo med, Ottobrunn, Germany). Ventilatory and gas exchange parameters were collected breath-by-breath using a metabolic measurement system (Metalyzer 3B-R3, Cortex Biophysik, Leipzig, Germany). The achievement of VO_2max_ was confirmed if ≥2 of the following criteria were met: Plateau in oxygen consumption, ≥90% of age-predicted maximum heart rate (HR_max_), peak respiratory exchange ratio ≥1.10, or a Borg scale rating of ≥19.^41^ Submaximal performance was assessed based on the power output at the ventilatory threshold (W_VT_), determined via the V-slope method.^42^

### 2.3 Dietary assessment and nutritional counseling

In the weeks preceding T−1 and T−2, patients completed 3-day food records using a standardized protocol (Nutri-Science, Freiburg, Germany). Records were analyzed using a specific software (PRODI 6 expert, Nutri-Science, Freiburg, Germany) to determine mean daily energy and macronutrient intakes. Based on the results, certified dietitians provided individualized counseling. Following international obesity management guidelines, patients were advised to achieve a daily caloric deficit of ~500 kcal relative to their habitual energy intake,^43^ and consume ≥1.0 g protein/kg body weight/day to preserve muscle mass.^44^ To support adherence and facilitate the practical application of dietary guidance, patients received recipe suggestions and categorized food lists.

### 2.4 Exercise intervention protocols

The training sessions were implemented at the exercise physiology laboratory of our research facility under the supervision of certified therapists to ensure safety and adherence to proper training techniques. Training was performed over a 12-week period, with two weekly sessions (total: 24 sessions). Sessions could be flexibly booked, provided a minimum of 24 hours recovery between sessions was maintained. Depending on group allocation, patients were assigned to one of the two exercise modalities: (1) HIGH-EX, consisting of LOW-HIIT combined with progressive 1-RT, or (2) MOD-EX, involving LOW-MIIT combined with moderate-progressive 1-RT.

The low-volume interval training protocol was conducted on cycle ergometers according to previous work by Reljic et al.^45^ Each interval training included a 2-minute warm-up, followed by five 1-minute exercise intervals interspersed with 1-minute low-intensity active recovery periods. A 3-minute cool-down phase concluded each interval training. Heart rate was continuously monitored using chest-strap sensors (acentas, Hörgertshausen*,* Germany), and real-time feedback on a screen was used to adjust resistance/cadence to meet individual intensity targets. Training intensity in HIGH-EX was progressively increased across the 12-week program as follows: Week 1–4: 80–85% HR_max_; week 5–8: 85–90% HR_max_; week 9–12: 90–95% HR_max_). In MOD-EX, the training intensity was constantly limited to 65–79% HR_max_. Total duration for interval training, including warm-up and cool-down, was 14 minutes.

Single-set resistance training consisted of five exercises targeting the major muscle groups using specific strength machines: Chest press, lat pulldown, lower back, abdominal crunch, and leg press (TechnoGym, Neu-Isenburg, Germany). In HIGH-EX, training loads progressed as follows: Week 1–4: 50–60% of 1-repetition maximum (1-RM), 15–20 repetitions; week 5–8: 60–70% 1-RM, 10–15 repetitions; and week 9–12: 70–80% 1-RM, 8–12 repetitions. In MOD-EX, training loads were limited to 50–60% 1-RM for 15–20 repetitions throughout the intervention period. Two minutes of rest were provided between exercises. Exercise loads were individually prescribed based on 1-RM assessments, which were conducted at T−1 and subsequently every four weeks throughout the intervention period, as previously described in detail.^32^ Total duration for 1-RT was ~20 minutes, resulting in a total session duration of ~34 minutes and a weekly exercise time of ~68 minutes, respectively.

#### 2.4.1 Blood lactate sampling

Capillary blood lactate concentrations were measured once weekly throughout the 12-week intervention period to monitor exercise intensity. Sampling was performed during the third interval of each training session, under standardized conditions. Specifically, the earlobe was punctuated using single-use safety lancets (Safety Lancet Normal, Sarstedt, Nümbrecht, Germany) after thorough disinfection with 70% isopropyl alcohol. The first drop of blood was wiped away to minimize contamination, and subsequently 20 µL of blood was collected using heparinized capillary tubes. The blood samples were immediately analyzed using an enzymatic-amperometric biosensor method (LabTrend lactate analyzer, BST Bio Sensor Technology, Holzheim, Germany). The system features a built-in calibration and quality control mechanism to ensure measurement stability and accuracy (CV of <3.0% for intra-assay precision).

### 2.5 Statistical analysis

All statistical procedures were conducted using SPSS software, version 24.0 (IBM Corp., Armonk, NY, USA). Descriptive data are presented as means ± standard deviation (SD), and changes from T−1 to T−2 are reported with corresponding 95% confidence intervals (CI). Normality of data distribution was assessed using the Shapiro-Wilk test, and homogeneity of variance was verified via the Levene’s test. A two-way repeated-measures analysis of variance (ANOVA) with a 3 × 2 design was employed to evaluate main and interaction effects. Where significant effects were identified, one-way ANOVAs with Holm–Sidak post-hoc corrections were used for between-group comparisons, and paired t-tests were performed to examine within-group changes. For variables displaying non-normal distributions, log or square-root transformations were applied to meet assumptions of parametric testing. If transformation failed to normalize the data (hsCRP), non-parametric methods were applied. Specifically, a Friedman’s two-way ANOVA by ranks was conducted, followed by Kruskal-Wallis tests with Dunn–Bonferroni corrections for between-group comparisons, and Wilcoxon signed-rank tests for within-group analyses. To account for potential trial-level variability, additional analyses including ‘trial’ as a covariate in all models were performed for the primary outcomes. Furthermore, sensitivity analyses were conducted by restricting comparisons of the exercise groups (from trial 1) to the control groups from trial 2 and trial 3 separately. Effect sizes were quantified using partial eta squared (η²) for ANOVA tests (interpreted as: small ≥0.01, medium ≥0.06, and large ≥0.14) and Kendall’s coefficient of concordance (W) for Friedman analyses (small ≥0.10, medium ≥0.30, large ≥0.50).^46^ To investigate the relationship between exercise intensity and exercise adaptations, Pearson’s correlation coefficients (r) were calculated between mean lactate values reached during the interval training and pre-/post-intervention changes of SCFA concentrations. Statistical significance was set at *p* < 0.05 for all analyses.

## 3 Results

### 3.1 Study flow

Across the three RCTs, 186 patients were randomized to either an exercise intervention (trial 1: *n* = 116) or a control group (trial 2: *n* = 40; trial 3: *n* = 30). Of these, 149 consented to provide stool samples for additional SCFA analysis. During the 12-week intervention, 34 patients discontinued for reasons detailed elsewhere.^32-34^ Two additional cases were excluded due to invalid or insufficient stool samples, resulting in a final analytic sample of *n* = 113. As no significant sex-related differences were found, data were pooled across sexes. Baseline characteristics of the included patients are summarized in Table 1.

**Table 1.** Patients’ baseline characteristics.

Variable	HIGH-EX	MOD-EX	CON
Age (years)	52.6 ± 12.4	53.5 ± 10.9	53.4 ± 16.9
Sex (male/female)	16/19	14/18	17/29
Microbial metabolites			
Total SCFAs (µmol/g)	136 ± 41	180 ± 56	175 ± 55
Acetate (µmol/g)	78 ± 22	101 ± 29	99 ± 31
Propionate (µmol/g)	30 ± 10	40 ± 17	38 ± 15
Butyrates (µmol/g)	28 ± 13	39 ± 16	37 ± 15
**Anthropometric variables**			
Body weight (kg)	114.3 ± 21.4	114.1 ± 23.8	116.5 ± 18.1
Body mass index (kg/m^2^)	38.3 ± 5.6	40.1 ± 6.0	39.0 ± 5.7
Body fat (%)	44.5 ± 6.4	45.1 ± 6.9	44.3 ± 5.2
Fat free mass (kg)	63.3 ± 13.4	62.5 ± 14.1	60.4 ± 7.2
Waist circumference (cm)	115 ± 13	117 ± 14	120 ± 18
**Blood pressure**			
Systolic blood pressure (mmHg)	134 ± 14	137 ± 15	133 ± 9
Diastolic blood pressure (mmHg)	87 ± 8	87 ± 13	93 ± 11
Mean arterial blood pressure (mmHg)	103 ± 8	103 ± 12	107 ± 8
**Clinical chemistry**			
Glucose (mg/dL)	107 ± 18	112 ± 25	105 ± 15
Triglycerides (mg/dL)	132 ± 66	150 ± 60	177 ± 57
HDL (mg/dL)	52 ± 11	53 ± 11	47 ± 7
LDL (mg/dL)	138 ± 26	148 ± 43	117 ± 27
hsCRP (mg/dL)	4.9 ± 6.1	4.6 ± 3.4	6.5 ± 4.1
**MetS z-score**	2.6 ± 2.9	3.4 ± 3.7	4.2 ± 3.2
**CPET variables**			
VO_2max_ (L)	2.38 ± 0.68	2.24 ± 0.63	2.16 ± 0.26
VO_2max_ (mL/kg/min)	21.3 ± 5.9	20.3 ± 6.2	18.8 ± 3.3
W_max_ (W)	169 ± 40	156 ± 44	163 ± 23
W_VT_ (W)	72 ± 15	73 ± 16	69 ± 7

Note: All data are presented as mean ± SD. Abbreviations: SCFAs = short chain fatty acids; HDL = high-density lipoprotein cholesterol; LDL = low-density lipoprotein cholesterol; hsCRP = high-sensitivity C-reactive protein; MetS z-score = metabolic syndrome severity score; CPET = cardiopulmonary exercise testing; VO2max = maximal oxygen uptake; Wmax = maximal power output; WVT = power output at the ventilatory threshold.

### 3.2 Microbial metabolites

**Figure 1.**
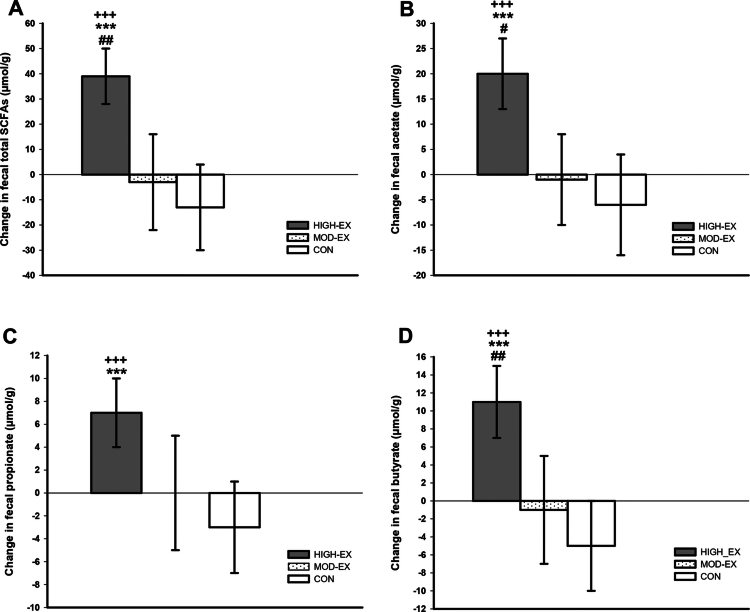
Changes in (A) total fecal SCFAs, (B) fecal acetate, (C) fecal propionate, and (D) fecal butyrate following the 12-week intervention. ^+++^(*p* < 0.001) denotes significant change between T−1 and T−2; ***(*p* < 0.001) denotes significant difference compared to CON; ^#^(*p* < 0.05), ^##^(*p* < 0.01) denotes significant difference compared to MOD-EX.

A significant group-by-time interaction was identified for total SCFAs (*p* < 0.001, η² = 0.17), butyrate (*p* < 0.001, η² = 0.18), acetate (*p* < 0.001, η² = 0.12), and propionate (*p* = 0.012, η² = 0.08). Post-hoc tests revealed significant increases in total SCFAs (+39 µmol/g, [95% CI: 27, 50 µmol/g], *p* < 0.001), acetate (+20 µmol/g, [95% CI: 13, 27 µmol/g], *p* < 0.001), propionate (+7 µmol/g, [95% CI: 3, 11 µmol/g], *p* < 0.001), and butyrate (+11 µmol/g, [95% CI: 7, 16 µmol/g], *p* < 0.001) in HIGH-EX, while no significant changes were observed in the two other groups. Compared to MOD-EX and CON, the increases in SCFAs (*p* = 0.003 and *p* < 0.001, respectively), acetate (*p* = 0.018 and *p* < 0.001, respectively), and butyrate (*p* = 0.003 and *p* < 0.001, respectively) were significantly greater in HIGH-EX. Moreover, the increase in propionate in HIGH-EX was larger compared to CON (*p* = 0.011) (Figure 1).

Including ‘trial’ as a covariate did not change the results, as ‘trial’ had no significant effect on any microbial metabolite outcome, and all group × time interactions remained significant. Sensitivity analyses using only one control trial at a time confirmed all main findings, except for propionate, which did not reach significance when comparing the exercise groups with CON from trial 3 only. This isolated result likely reflects reduced statistical power in the restricted sample. Overall, these analyses support the robustness of our findings across trials.

### 3.3 Anthropometric variables

Significant main effects of time were observed for body weight (*p* = 0.039, η² = 0.06) and BMI (*p* = 0.003, η² = 0.11). Further, a significant group-by-time interaction was found for body weight (*p* = 0.011, η² = 0.12), BMI (*p* = 0.018, η² = 0.10), body fat (*p* = 0.005, η² = 0.13), and WC (*p* = 0.002, η² = 0.15). Only HIGH-EX significantly reduced body weight and body fat. Reductions in body weight (*p* = 0.008), BMI (*p* = 0.017), and body fat (*p* = 0.004) were significantly greater in HIGH-EX compared to MOD-EX. Waist circumference decreased significantly in HIGH-EX and MOD-EX but not in CON. Compared to CON, the reductions in WC were significantly larger in HIGH-EX (*p* < 0.001) and MOD-EX (*p* = 0.007) (Table 2).

**Table 2.** Changes (*Δ*) in patients’ anthropometric and cardiometabolic data between T−1 and T−2.

Variable	HIGH-EX	MOD-EX	CON
**Anthropometric variables**			
Body weight (kg)	–3.7 [–5.9, –1.6]^##^	–0.4 [–1.5, 2.3]	–1.3 [–2.6, 0.1]
*p*-value	<0.001	0.660	0.058
Body mass index (kg/m^2^)	–1.2 [–1.9, –0.5]^#^	–0.2 [–0.5, 0.1]	–0.4 [–1.0, 0.2]
*p*-value	<0.001	0.177	0.199
Body fat (%)	–1.7 [–2.9, –0.6]^##^	–0.3 [–0.2, 0.8]	–0.2 [–0.8, 0.4]
*p*-value	0.002	0.263	0.556
Fat free mass (kg)	–0.7 [–1.6, 0.1]	–0.9 [–1.6, –0.1]	–0.4 [–0.9, 0.2]
*p*-value	0.083	0.021	0.145
Waist circumference (cm)	–3.5 [–5.3, –1.8]***	–1.9 [–3.3, –0.5]**	2 [–2.0, 8.0]
*p*-value	<0.001	0.012	0.229
**Blood pressure**			
Systolic blood pressure (mmHg)	–6 [–11, –2]*	–6 [–11, –1]*	7 [2, 12]
*p*-value	0.005	0.006	0.008
Diastolic blood pressure (mmHg)	–4 [–7, –1]	–4 [–9, 0]	0 [–5, 5]
*p*-value	0.006	0.023	0.968
Mean arterial blood pressure (mmHg)	–5 [–8,–2]*	–5 [–8, –1]*	3 [2, 5]
*p*-value	0.001	0.006	<0.001
**Clinical chemistry**			
Glucose (mg/dL)	1 [–3, 4]	0 [–3, 3]	–2 [–4, 0]
*p*-value	0.691	0.902	0.067
Triglycerides (mg/dL)	–4 [–27, 18]	8 [–12, 28]	13 [–26, 51]
*p*-value	0.693	0.427	0.480
HDL (mg/dL)	1 [–1, 3]	1 [–2, 3]	–4 [–5, 1]
*p*-value	0.472	0.495	0.139
LDL (mg/dL)	1 [–5, 6]	–4 [–11, 3]	9 [–12, 30]
*p*-value	0.820	0.220	0.355
hsCRP (mg/dL)	–2.2 [–4.1, 0.3]	–1.3 [–2.0, 0.7]	–0.8 [–3.5, 1.9]
*p*-value	<0.001	<0.001	0.526
**MetS z-score**	–1.1 [–1.8, –0.3]**	–0.5 [–1.1, 0.1]	1.0 [0.2, 1.8]
*p*-value	0.008	0.045	0.025

Note: All data are presented as mean changes and respective 95% CIs in brackets. *P*-values below *Δ*-values denote significant within-group changes; *(*p* < 0.05), **(*p* < 0.01), ***(*p* < 0.001) denote significant difference vs. CON; ^#^(*p* < 0.05), ^##^(*p* < 0.01) denote significant difference vs. MOD-EX. Abbreviations: HDL = high-density lipoprotein cholesterol; LDL = low-density lipoprotein cholesterol; hsCRP = high-sensitivity C-reactive protein; MetS z-score = metabolic syndrome severity score.

### 3.4 Cardiometabolic variables and inflammation

Significant group-by-time interactions were detected for systolic BP (*p* = 0.009, η² = 0.12), MAB (*p* = 0.016, η² = 0.11), and the MetS z-score (*p* = 0.009, η² = 0.12). Further, a significant main effect of time was found for diastolic BP (*p* = 0.035, η² = 0.06) and hsCRP (*p* = 0.011, W = 0.31). Post-intervention systolic/diastolic BP and MAB were found to be reduced in both HIGH-EX and MOD-EX. Compared to CON, reductions in systolic BP and MAB were significantly larger in HIGH-EX (*p* = 0.021 and *p* = 0.016, respectively) and MOD-EX (*p* = 0.024 and *p* = 0.029, respectively). Both, HIGH-EX and MOD-EX experienced significant reductions hsCRP. Furthermore, post hoc tests revealed a significant decrease of MetS z-score in HIGH-EX and MOD-EX from T−1 to T−2. The improvements in the MetS z-score were significantly larger in HIGH-EX compared to CON (*p* = 0.007) (Table 2).

### 3.5 Physical fitness variables

Significant main effects of time and group-by-time interactions were found for relative VO_2max_ (*p* < 0.001, η² = 0.49 and *p* < 0.001, η² = 0.44, respectively), absolute VO_2max_ (*p* < 0.001, η² = 0.26 and *p* < 0.001, η² = 0.33, respectively), maximum power output (W_max_, *p* < 0.001, η² = 0.33 and *p* < 0.001, η² = 0.26, respectively) and W_VT_ (*p* < 0.001, η² = 0.31 and *p* = 0.006, η² = 0.13, respectively). Both exercise groups improved relative and absolute VO_2max_ and W_max_ to a significantly larger extent compared to CON (all *p*-values < 0.001). Additionally, increases in W_VT_ were significantly greater in HIGH-EX compared to CON (*p* = 0.006) (Table 3).

**Table 3.** Changes *(Δ)* in patients’ CPET data between T−1 and T−2.

Variable	HIGH-EX	MOD-EX	CON
VO_2max_ (L)	0.41 [0.31, 0.50]***	0.32 [0.22, 0.43]***	–0.18 [–0.28, –0.08]
*p*-value	<0.001	<0.001	0.003
VO_2max_ (mL/kg/min)	4.2 [3.5, 4.9]***	2.7 [2.1, 3.3]***	–0.7 [–1.3, –0.2]
*p*-value	<0.001	<0.001	0.018
W_max_ (W)	25 [20, 30]***	19 [12, 26]***	–5 [–8, –2]
*p*-value	<0.001	<0.001	0.006
W_VT_ (W)	22 [15, 28]**	14 [9, 19]	2 [–4, 8]
*p*-value	<0.001	<0.001	0.432

Note: All data are presented as mean changes and respective 95% CIs in brackets. *P*-values below *Δ*-values denote significant within-group changes; **(*p* < 0.01), ***(*p* < 0.001) denote significant difference vs. CON. Abbreviations: VO2max = maximal oxygen uptake, Wmax = maximal power output, WVT = power output at the ventilatory threshold.

There were significant time effects for 1-RM of chest (*p* < 0.001, η² = 0.59), upper back (*p* < 0.001, η² = 0.62), abdominals (*p* < 0.001, η² = 0.50), lower back (*p* = 0.001, η² = 0.44) and legs (*p* < 0.001, η² *=* 0.39). Both, HIGH-EX and MOD-EX improved 1-RM values in all major muscle groups (ranging from 19–26%, and 22–29%, respectively, all *p* < 0.001) (Table 4). Given that 1-RM testing in CON was only conducted in trial 3, a pooled representation of strength values for both CON groups was not possible.

**Table 4.** One-repetition maximum strength values in HIGH-EX and MOD-EX at T−1 and T−2.

	HIGH-EX		MOD-EX	
Variable	T−1	T−2	T−1	T−2
1-RM chest (kg)	38 ± 18	45 ± 19^+++^	35 ± 17	45 ± 19^+++^
1-RM upper back (kg)	52 ± 17	62 ± 22^+++^	50 ± 17	62 ± 18^+++^
1-RM abdominals (kg)	32 ± 11	38 ± 14^+++^	27 ± 13	34 ± 13^+++^
1-RM lower back (kg)	57 ± 19	73 ± 30^+++^	57 ± 25	75 ± 36^+++^
1-RM legs (kg)	132 ± 34	162 ± 55^+++^	129 ± 53	167 ± 83^+++^

Note: All data are presented as mean ± SD. *P*-values denote significant difference vs. T-2; ^+++^(*p* < 0.001). Abbreviations: 1-RM = one-repetition maximum strength.

### 3.6 Training data, adverse events and dietary intakes

The targeted exercise intensities during interval training were successfully reached in both exercise groups, as demonstrated by the mean peak heart rates attained during the intervals. HIGH-EX patients achieved an average of 95 ± 4% of their individual HR_max_, while those in MOD-EX reached 78 ± 7% HR_max_. Mean lactate concentration measured during the third interval bout was 8.7 ± 2.2 mmol/L in HIGH-EX, which was significantly higher (*p* < 0.001) compared to MOD-EX (4.1 ± 0.7 mmol/L). Further, all patients reached/maintained their prescribed resistance loads during 1-RT throughout the 12-week intervention in accordance with the protocol described in Section 2.4. Exercise adherence was high in both exercise groups, with mean attendance rates of 94 ± 9% in HIGH-EX and 93 ± 8% in MOD-EX. Notably, the data set of both exercise groups consisted of patients assigned to two different training sequences (i.e., LOW-HIIT/LOW-MIIT followed by 1-RT or vice versa), since analyzing order effects was one objective of the original trial.^34^ Results of the original trial demonstrated no significant order effects on key physiological outcomes, including cardiometabolic markers, inflammation and fitness variables. A subgroup analysis of the present study (data not shown) confirmed the comparability of the two exercise sequences with respect to effects on SCFAs. Therefore, pooling data of both exercise orders was considered methodologically justified and allowed for increased statistical power without introducing systematic bias related to exercise sequence.

Only a few minor adverse events potentially attributable to the exercise interventions were reported in HIGH-EX, including mild knee pain (*n* = 3), mild back pain (*n* = 1), and mild shoulder pain (*n* = 1).

Significant main effects of time were found for daily energy (*p* < 0.001, η² = 0.31), protein (*p* < 0.001, η² = 0.14), fat (*p* < 0.001, η² = 0.31), and carbohydrate (CHO, *p* < 0.001, η² = 0.28) intakes. All groups significantly (*p* < 0.001) reduced energy intake. Energy restriction was mainly achieved through a reduction in CHO (HIGH-EX: *p* = 0.001; MOD-EX: *p* < 0.001; CON: *p* = 0.008) and fat intake (HIGH-EX: *p* < 0.001; MOD-EX: *p* < 0.001; CON: *p* = 0.016). Protein intake increased in all groups (HIGH-EX: *p* = 0.030; MOD-EX: *p* < 0.001; CON: *p* = 0.014). Fiber intake showed no significant changes over time (*p* = 0.105), no between-group differences (*p* = 0.344), and no interaction effects (*p* = 0.766) (Table 5).

**Table 5.** Dietary intakes in HIGH-EX, MOD-EX and CON at T−1 and T−2.

	HIGH-EX		MOD-EX		CON	
Variable	T−1	T−2	T−1	T−2	T−1	T−2
Energy intake (kcal)	2418 ± 740	1764 ± 489^+++^	2598 ± 1032	1764 ± 489^+++^	2244 ± 540	1812 ± 553^+++^
Protein (g/kg)	1.1 ± 1.1	1.5 ± 0.6^+^	0.9 ± 0.3	1.4 ± 0.5^+++^	0.8 ± 0.2	1.3 ± 0.3^+^
Fat (g/kg)	0.9 ± 0.3	0.6 ± 0.2^+++^	1.0 ± 0.5	0.7 ± 0.3^+++^	0.7 ± 0.3	0.5 ± 0.2^+^
Carbohydrates(g/kg)	2.1 ± 0.7	1.7 ± 0.6^++^	2.4 ± 0.8	1.7 ± 0.5^+++^	2.1 ± 0.4	1.5 ± 0.5^++^
Fiber (g)	23 ± 13	22 ± 9	26 ± 13	24 ± 9	20 ± 7	18 ± 6

Notes: All data are presented as mean ± SD. P-values denote significant difference vs. T-2; ^+^(*p* < 0.05), ^++^(p < 0.01), ^+++^(p < 0.001).

### 3.7 Relationship between training intensity and changes in microbial metabolites

**Figure 2.**
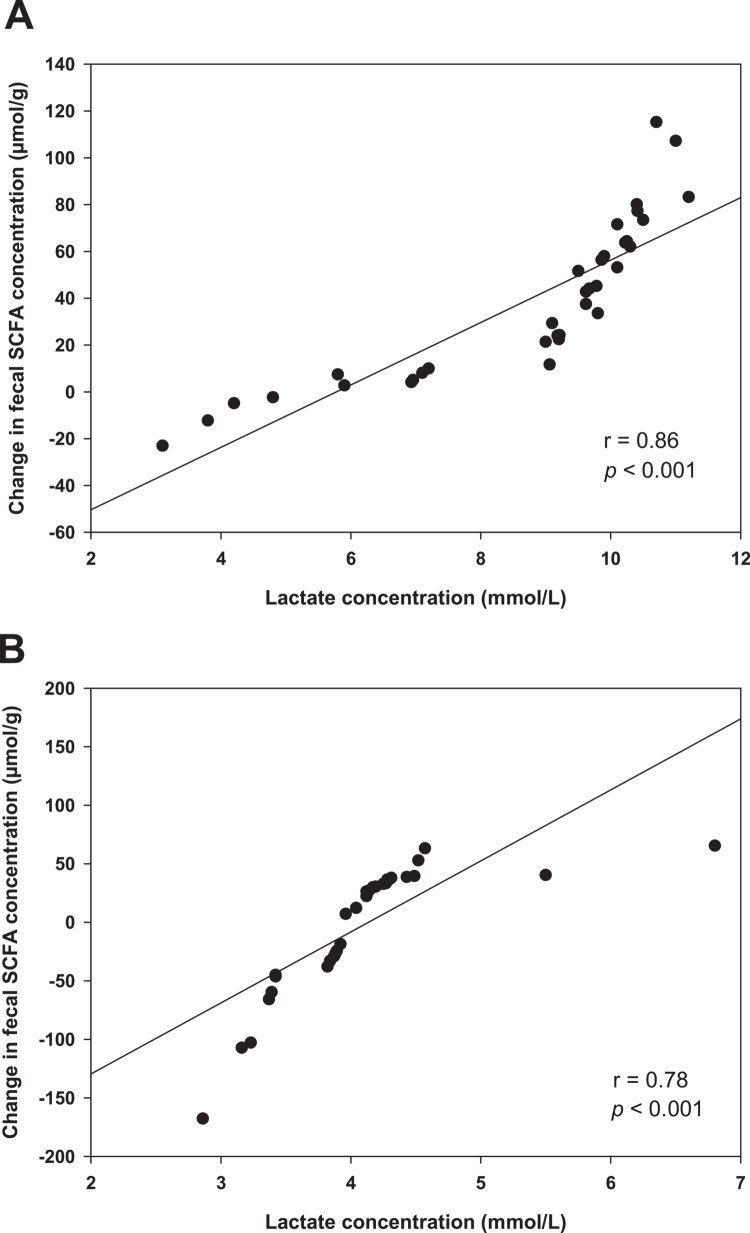
Correlation between mean lactate concentration during interval training and change in SCFA concentration in (A) HIGH-EX, and (B) MOD-EX.

Across the entire exercise sample, mean lactate levels attained during interval training were significantly correlated with changes in total SCFAs (r = 0.68, *p* < 0.001), acetate (r = 0.61, *p* < 0.001), propionate (r = 0.50, *p* < 0.001), and butyrate (r = 0.59, *p* < 0.001). When analyzed separately, these associations remained significant in both groups. In HIGH-EX, lactate levels were significantly associated with changes in total SCFAs (r = 0.86, *p* < 0.001), acetate (r = 0.76, *p* < 0.001), propionate (r = 0.62, *p* < 0.001), and butyrate (r = 0.60, *p* < 0.001). In MOD-EX, lactate concentrations showed correlations with total SCFAs (r = 0.78, *p* < 0.001), acetate (r = 0.68, *p* < 0.001), propionate (r = 0.71, *p* < 0.001), and butyrate (r = 0.65, *p* < 0.001) (Figure 2).

## 4 Discussion

This is the first study to systematically examine how exercise intensity affects fecal SCFA profiles in obese MetS patients. Both HIGH-EX and MOD-EX improved physical fitness, cardiometabolic risk, and low-grade inflammation. However, only HIGH-EX significantly increased fecal SCFAs, including acetate, propionate, and butyrate. These observations support the hypothesis that exercise intensity appears to serve as a key modulator of gut microbial metabolite responses.^21^^,^^22^

Our findings align with and extend previous work. Allen et al.,^19^ for example, reported that moderate-intensity aerobic training increased fecal SCFA levels in lean but not in obese individuals, suggesting that exercise-induced alterations in gut microbial metabolites may be dependent on obesity status. Liu et al.^18^ demonstrated increases in fecal propionate concentration following higher-intensity training in overweight and obese men, highlighting the importance of training intensity for notable modifications in intestinal metabolites. More recently, Torquati et al.^20^ found that exercise intensity differentially affected the abundance of specific microbial taxa in patients with type 2 diabetes, supporting the notion that intensity plays a critical role in shaping gut microbiome adaptations. By extending these insights to a MetS cohort and systematically contrasting high- versus moderate-intensity exercise, our study provides novel and clinically relevant evidence that higher-intensity exercise may be required to elicit meaningful increases in microbial SCFA production in populations at elevated cardiometabolic risk.

The mechanistic underpinnings of the SCFA response to exercise intensity remain an area of active investigation. One plausible hypothesis is that lactate metabolism may act as an important mediator of these effects. Prior studies have proposed a possible "lactate–SCFA link", suggesting that exercise-induced lactate production in skeletal muscle could serve as a substrate for specific bacterial taxa capable of converting lactate into SCFAs.^47-49^ While our observed correlation between mean training lactate levels and SCFA changes is consistent with this hypothesis, we cannot establish causality, and this topic warrants further investigation.

In particular, butyrate showed the highest relative increase following HIGH-EX. This SCFA is primarily produced via the acetyl-CoA pathway by anaerobic, glucose-fermenting bacteria. Species such as *Anaerostipes spp*. and *Eubacterium hallii* can convert lactate into butyrate under anaerobic conditions, potentially linking host lactate production to microbial butyrogenesis.^50^ Thus, the observed rise in butyrate likely may reflect both increased lactate availability and enhanced colonization or activity of these lactate-utilizing bacteria. Likewise, propionate is produced through multiple microbial pathways, including the succinate, acrylate, and propanediol routes. Bacteria from the *Bacteroidetes phylum* primarily use the succinate route, while *Firmicutes*, most notably like *Veillonella spp*. utilize the lactate-dependent acrylate pathway.^51^ The rise in propionate following HIGH-EX may reflect elevated lactate flux favoring acrylate-mediated synthesis, although this interpretation remains speculative.

Acetate, the most abundant SCFA, is produced by a broad array of microbial taxa and supports energy metabolism and butyrate synthesis. Its increase in the HIGH-EX group may reflect enhanced microbial fermentation, alongside systemic changes induced by repeated intense exercise, such as improved gut perfusion and increased luminal substrate availability, that create a favorable environment for acetate-producing microbes.^52^

Our findings align with evidence identifying SCFAs as key microbial metabolites supporting metabolic health.^7^^,^^8^ SCFA increases after HIGH-EX may be clinically relevant for MetS patients, particularly the rise in butyrate, which enhances gut barrier integrity, modulates inflammation, and regulates host energy metabolism via histone deacetylase inhibition and G-protein coupled receptor (GPCR) activation.^53^ Additionally, acetate and propionate play complementary roles, with acetate having an important function for energy and substrate metabolism,^54^ while propionate has been implicated in hepatic gluconeogenesis and satiety signaling via the “gut–brain axis”.^55^ Moreover, elevated SCFA levels have been linked to improved muscle health and performance.^56^ Consequently, from a clinical perspective, our findings suggest that low-volume, higher-intensity exercise may confer not only improvements in physical fitness and traditional cardiometabolic biomarkers, but also beneficial alterations in gut metabolites. This supports the use of such training protocols in obesity and MetS care, particularly given time constraints often reported in these populations.^27^

While HIGH-EX showed the most robust physiological benefits, MOD-EX also significantly improved cardiometabolic risk indices, CRF, and muscle strength. Although trends favored HIGH-EX, differences between groups were not statistically significant. Additionally, hsCRP significantly decreased in both exercise groups, further emphasizing the anti-inflammatory potential of structured physical activity as reported previously.^28^^,^^57^ These results highlight moderate-intensity, low-volume exercise as an effective option to improve cardiometabolic health, particularly for those who are physically deconditioned, or otherwise unable/unwilling to engage in higher-intensity training. In such cohorts, MOD-EX could serve as a preparatory strategy to build foundational fitness and tolerance before progressing to more demanding exercise regimens. From a mechanistic standpoint, gut-derived SCFAs may be determining in mediating some of these cardiometabolic changes. In HIGH-EX, increases in acetate and butyrate may have supported BP reductions via GPCR activation, sympathetic nervous system modulation, and enhancement of vascular function.^58^ Butyrate’s anti-inflammatory effects, mainly linked to nuclear factor kappa B (NF-κB) suppression and promotion of regulatory T-cell populations,^59^ could also explain reduced inflammation. However, the lack of SCFA changes in MOD-EX suggests that other exercise-driven mechanisms, such as improved endothelial function^60^ or reductions in adipose tissue,^61^ likely contributed to the improvements in BP and inflammation. Furthermore, HIGH-EX and MOD-EX produced clinically meaningful gains in VO_2max_^62^ and muscle strength,^63^ supporting the efficacy of both, higher- and moderate-intensity exercise protocols as health-promoting strategies. In contrast, CON showed no significant changes, underscoring the limited impact of caloric restriction alone without exercise on cardiometabolic and fitness outcomes.

Notably, all groups achieved similar dietary changes. As intended, energy restriction was primarily driven by decreases in CHO and fat consumption, alongside modest increases in protein intake in HIGH-EX, MOD-EX, and CON. Importantly, fiber intake remained constant in all groups, providing a critical point of control when interpreting the SCFA outcomes. Thus, the SCFA increases seen only in HIGH-EX are unlikely diet-driven and instead point to exercise intensity as a key factor modulating microbial fermentation and metabolite production.

### 4.1 Limitations and strengths

While this study provides novel and clinically meaningful insights into the interplay between exercise intensity, gut microbial metabolites, and cardiometabolic health, some limitations should be acknowledged. First, the analysis builds on data derived from three previously conducted RCTs. While the decision to pool the data from different original studies was considered methodologically justified, it still introduces an added layer of complexity and potential heterogeneity across trials must be considered. However, both exercise groups originated from the same trial with identical design and protocols, and the two control groups were highly comparable in terms of intervention (standard care), participant characteristics, study staff, and laboratory handling. Importantly, all procedures and measurements were performed in a well-controlled laboratory setting with stable ambient conditions and stool sample handling was strictly standardized. Additional analyses confirmed the robustness of our findings: ‘Trial’ as a covariate did not alter the results, and sensitivity analyses restricting the control group to either trial 2 or trial 3 yielded consistent findings across outcomes, with the exception of propionate when only trial 3 was included. This isolated result likely reflects reduced statistical power in the restricted sample rather than a true divergence. Taken together, these results strengthen confidence that the observed effects reflect genuine intervention effects rather than trial-specific variability.

Second, although stool samples were collected using standardized test kits and transported to the laboratory within 24–48 h—well within the validated stability range (≥5 days) declared by the analytics provider—we acknowledge that short-term storage under non-frozen conditions may not fully suppress microbial activity. This may have introduced a degree of variability. However, as all groups and time points followed the same standardized collection and shipping procedures, any potential bias is unlikely to explain the systematic group × time effects observed.

Third, while SCFA levels provide meaningful insights into gut activity, microbial composition was not directly assessed. Including taxonomic data obtained through sequencing would have clarified which bacterial populations contributed to SCFA shifts and their link to host responses, limiting mechanistic interpretation. Fourth, the use of fecal SCFAs as biomarkers, while valid, is inherently limited in that these concentrations reflect unabsorbed SCFAs remaining in the gut lumen rather than systemic exposure. Given that the majority of SCFAs are rapidly absorbed and utilized by peripheral tissues, fecal levels may substantially underestimate total production. Accordingly, future studies should incorporate measures of circulating SCFAs and, ideally, receptor expression and signaling activity to more directly assess the systemic relevance and mechanistic pathways of exercise-induced changes in SCFA metabolism.

Fifth, dietary intake data, although collected using validated protocols, were based on self-report and thus susceptible to underreporting or recall bias. Finally, the intervention duration of 12 weeks, though sufficient to elicit significant changes in SCFAs and key cardiometabolic parameters, does not address long-term sustainability or clinical translation. Extended follow-up is needed to determine if microbial and metabolic effects persist and translate into lasting health benefits.

Despite limitations, this study has several strengths. Most notably, it is the first to directly compare exercise intensity effects on SCFAs in a clinical population. The use of individualized exercise intensity prescriptions, regularly controlled based on lactate measurements, ensures physiological precision in training load across groups. Moreover, the integration of gut microbial metabolites with cardiometabolic, inflammatory, and physical fitness parameters offers a multidimensional view of the systemic adaptations elicited by exercise.

### 4.2 Conclusions and perspectives

Our results demonstrate that exercise intensity plays a key role in modulating gut microbial metabolites, with only higher-intensity training elevating fecal SCFA levels in obese MetS patients. These increases correlated with lactate responses, suggesting a potential metabolic link that should be further examined in future mechanistic studies. Both HIGH-EX and MOD-EX improved physical fitness, inflammation, and cardiometabolic health, with no major group-differences in most clinical outcomes, supporting moderate-intensity exercise as a viable option for deconditioned individuals. Caloric restriction alone (CON) did not significantly improve SCFAs or metabolic health, emphasizing the central role of exercise in clinical lifestyle interventions. Personalized exercise prescriptions targeting the gut-muscle-metabolism axis may become key in future prevention and therapy.

## Data Availability

The data that support the findings of this study are available from the corresponding author upon reasonable request. The data are not publicly accessible because of information that could compromise the privacy or consent of research participants.

## Abbreviations

BMI: Body Mass Index
BP: Blood Pressure
CHO: Carbohydrates
CON: Control Group
CPET: Cardiopulmonary Exercise Test
CRF: Cardiorespiratory Fitness
CV: Coefficients Of Variation
HDL: High-Density Lipoprotein Cholesterol
HIGH-EX: Higher-Intensity Exercise
HR_max_: Maximum Heart Rate
hsCRP: High-Sensitivity C-Reactive Protein
HS-GC-MS: Headspace Gas Chromatography With Mass Spectrometry
LDL: Low-Density Lipoprotein Cholesterol
LOW-HIIT: Low-Volume High-Intensity Interval Training
LOW-MIIT: Low-Volume Moderate-Intensity Interval Training
MAB: Mean Arterial Blood Pressure
MetS: Metabolic Syndrome
MetS Z-Score: Metabolic Syndrome Severity Score
MOD-EX: Moderate-Intensity Exercise
RCTs: Randomized Controlled Trials
SCFAs: Short-Chain Fatty Acids
T-1: Baseline Assessments
T-2: Post-Intervention Assessments
VO_2max_: Maximal Oxygen Uptake
WC: Waist Circumference
W_max_: Maximum Power Output
W_VT_: Power Output At Ventilatory Threshold
1-RM: 1-Repetition Maximum
1-RT: Single-Set Resistance Training
